# Assessing the Influence of Proton Pump Inhibitors on Clinical Outcomes in Hormone Receptor-Positive Metastatic Breast Cancer Patients Receiving CDK4/6 Inhibitors: Evidence from a Ribociclib-Dominant Cohort

**DOI:** 10.3390/medicina61111960

**Published:** 2025-10-31

**Authors:** Enes Erul, Nejat Emre Öksüz, Erman Akkus, Pınar Kubilay Tolunay, Elif Berna Köksoy, Hatime Arzu Yasar

**Affiliations:** Division of Medical Oncology, Department of Internal Medicine, Ankara University Faculty of Medicine, Ankara 06620, Türkiye; nejatemreoksuz@gmail.com (N.E.Ö.); eakkus@ankara.edu.tr (E.A.); ptolunay@ankara.edu.tr (P.K.T.); koksoy@ankara.edu.tr (E.B.K.); hayasar@ankara.edu.tr (H.A.Y.)

**Keywords:** CDK4/6 inhibitors, proton pump inhibitors, drug–drug interactions, HR-positive/HER2-negative metastatic breast cancer, ribociclib, palbociclib, endocrine therapy, real-world observational study, progression-free survival, bioavailability

## Abstract

*Background and Objectives*: Endocrine therapy combined with CDK4/6 inhibitors are widely recognized as the standard first-line approach for treating hormone receptor-positive HER2-negative (HR+/HER2−) metastatic breast cancer (mBC). Nonetheless, potential pharmacokinetic interactions—particularly with proton pump inhibitors (PPIs)—have raised concerns about reduced drug bioavailability and compromised therapeutic efficacy. *Materials and Methods*: This retrospective analysis included 92 patients with HR+/HER2− mBC who received either ribociclib or palbociclib between 2019 and 2024 at a single tertiary care center. Patients were stratified according to whether they were concurrently using PPIs during CDK4/6 inhibitor treatment. The primary endpoint assessed was progression-free survival (PFS). The study population was dominated by ribociclib users, and the results primarily apply to ribociclib; the palbociclib analyses are descriptive only due to the very small numbers (*n* = 6). *Results*: The median PFS was significantly shorter in patients who received concomitant PPI therapy compared with those who did not (5.6 vs. 24.4 months; *p* < 0.001). Multivariable analysis identified PPI use, endocrine resistance, and the presence of three or more metastatic sites as independent predictors of reduced PFS. In the ribociclib-only cohort (*n* = 86), the association persisted (adjusted HR 6.36, 95% CI 3.02–13.37, *p* < 0.001). No notable differences in toxicity profiles were observed between the groups. *Conclusions*: In this ribociclib-dominant real-world cohort, concomitant PPI use was associated with shorter PFS, and the findings primarily apply to ribociclib. Given the potential for confounding by the indication/comorbidity inherent to retrospective studies, the results should be interpreted as associational. These data support the cautious use of non-essential PPIs during ribociclib therapy and underscore the need for prospective agent-specific pharmacokinetic studies.

## 1. Introduction

According to GLOBOCAN 2022 data, breast cancer was newly diagnosed in an estimated 2.3 million women globally and accounted for approximately 670,000 deaths [1]. Constituting nearly one-third of all cancer diagnoses in women, breast cancer continues to pose a major public health burden—particularly affecting younger female populations [2]. Epidemiological studies indicate that 5% to 10% of cases present with de novo metastatic disease, while 20% to 30% of patients initially diagnosed with early-stage breast cancer develop distant metastases over time [2,3]. Among those with metastatic breast cancer (mBC), the hormone receptor-positive HER2-negative (HR+/HER2−) subtype remains the most prevalent molecular profile [2,3].

Cyclin-dependent kinase 4/6 inhibitors (CDK4/6i) have emerged as a pivotal component in the frontline management of HR+/HER2− mBC [4]. Their integration with endocrine therapy (ET) has become the standard therapeutic approach, supported by robust evidence from multiple randomized trials demonstrating significant improvements in both overall survival (OS) and progression-free survival (PFS) [5,6,7,8,9,10]. Three oral CDK4/6 inhibitors are approved for this setting—ribociclib, palbociclib, and abemaciclib—with broadly comparable efficacies across pivotal trials when paired with ET. Notably, this therapeutic strategy has achieved outcomes comparable with, and in some instances surpassing, those of chemotherapy—particularly in patients without immediate risk of visceral crisis or organ dysfunction. Importantly, this benefit extends across clinical subgroups, regardless of whether the disease is de novo or recurrent and irrespective of menopausal status [5,6,7,8,9,10].

Despite their established therapeutic benefit in HR+/HER2− metastatic breast cancer, CDK4/6 inhibitors are subject to pharmacokinetic challenges due to their dependence on the CYP3A4 metabolic pathway, raising the risk of interaction with concomitant medications [11]. These interactions may significantly affect both the efficacy and toxicity profiles of CDK4/6 inhibitors, underscoring the importance of vigilant monitoring and judicious management of concomitant medications. Proton pump inhibitors—such as omeprazole, lansoprazole, and pantoprazole—are among the most commonly prescribed medications globally, primarily indicated for acid-associated gastrointestinal disorders, such as gastroesophageal reflux disease (GERD) and peptic ulcer pathology [12]. Given that CDK4/6 inhibitors are weak bases, concerns have been raised regarding their bioavailability in the context of sustained gastric acid suppression [13]. The prolonged elevation of gastric pH induced by long-term PPI use may potentially alter the absorption and systemic exposure of these agents, thereby diminishing their therapeutic efficacy [13]. However, the existing studies in the literature report conflicting results, as to whether the concurrent administration of PPIs significantly impacts the therapeutic efficacy of CDK4/6 inhibitors [14,15,16,17]. Del Re et al. reported that concomitant PPIs did not compromise outcomes in ribociclib-treated patients [16], whereas Eser et al. found a shorter PFS with PPI use in cohorts treated with ribociclib and palbociclib [18]. In light of these considerations, our study sought to evaluate whether the concomitant use of PPI impacted the prognosis in patients with mBC receiving palbociclib or ribociclib. Specifically, we investigated whether PPI co-administration altered the efficacy or toxicity profiles of CDK4/6 inhibitors in routine clinical settings.

## 2. Materials and Methods

Patients with HR+/HER2− mBC who received endocrine therapy plus a CDK4/6 inhibitor between 2019 and 2024 at our tertiary cancer center were retrospectively identified, including those receiving therapy in both first and subsequent lines. Patients were eligible for inclusion if they had accessible and complete records regarding concomitant medication use, including proton pump inhibitors. HR+/HER2− mBC was characterized by tumors exhibiting an estrogen receptor (ER) expression of 10% or higher and an HER2-negative status confirmed by immunohistochemistry (IHC) scores of 0 to 1+ or 2+ with a negative result on in situ hybridization (ISH) testing [19,20]. Patients were categorized based on concomitant PPI use during CDK4/6 inhibitor treatment. Patients were classified as having concomitant PPI use if a PPI was documented on more than 50% of days between CDK4/6 inhibitor initiation and discontinuation/last follow-up; those below this threshold were considered to not have concomitant PPI use. Exposure was ascertained from the electronic health record, using prescription/dispensation data and clinic notes. The records did not include the clock-time of administration for PPIs or CDK4/6 inhibitors, and there was no standardized documentation of counseling on dose separation during the study period. Consequently, we were unable to distinguish within-day concurrent from staggered dosing. Additionally, time zero was the CDK4/6 inhibitor initiation date. This summary relies on information accrued after time zero; hence, patients who started PPIs later necessarily contributed some event-free time before their first PPI day in the exposed category. We therefore interpret the comparisons as associational and discuss the likely direction of bias in the limitations.

Endocrine sensitivity was characterized by the lack of previous systemic treatment for metastatic disease or relapse occurring ≥12 months following the completion of adjuvant endocrine treatment. The definition of primary endocrine resistance included disease recurrence within 24 months after adjuvant ET or progression during the first 6 months of endocrine therapy administered for metastatic disease [21,22]. Clinical information pertinent to the study was obtained retrospectively from the hospital’s electronic record system. The variables comprised patient demographics, the date of initial diagnosis, menopausal status, the date of metastatic disease onset, the date of disease progression, the ECOG performance status, comorbidities, regularly used medications, pathological characteristics (including hormone receptor and HER2 status), the number and sites of metastases, and treatment-related adverse events. Safety and tolerability assessments of CDK4/6 inhibitors were conducted based on hematologic and non-hematologic toxicities, which were graded in accordance with the Common Terminology Criteria for Adverse Events (CTCAE), version 5.0 [23]. Hematologic toxicities—including neutropenia, anemia, and thrombocytopenia—as well as non-hematologic events such as liver toxicity, anemia, and QT interval prolongation were assessed. Particular attention was given to adverse events that led to treatment delays, dose reductions, or interruptions. For CDK4/6 inhibitors, each treatment cycle lasted 28 days. Ribociclib was initiated at a standard daily dose of 600 mg, while palbociclib was administered at 125 mg daily, both following a 21-days-on/7-days-off schedule (treatment on days 1–21, with rest on days 22–28 of each 28-day cycle). In premenopausal or perimenopausal women, CDK4/6 inhibitor therapy (either ribociclib or palbociclib) was coupled with a luteinizing hormone–releasing hormone (LHRH) agonist to ensure adequate ovarian suppression.

All analyses were performed in IBM SPSS Statistics for Windows, version 22.0 (IBM Corp., Armonk, NY, USA). Group comparisons of categorical and clinical variables were conducted, using either the Chi-square test or Fisher’s exact test, depending on the data characteristics and suitability. PFS was defined as the interval from the initiation of CDK4/6 inhibitor therapy to the earliest date of disease progression, death from any cause, or last follow-up. The effect of concomitant PPI use on PFS was evaluated using the log-rank test. Multivariate analyses were conducted through Cox proportional hazards regression with backward elimination, including variables significant in univariate testing. Kaplan–Meier estimates were used to generate survival curves, and a two-sided *p*-value < 0.05 was considered statistically significant. The overall response rate (ORR) was defined as the percentage of patients achieving complete or partial response, while the disease control rate (DCR) included a complete response, a partial response, and stable disease.

## 3. Results

**Figure 1:**
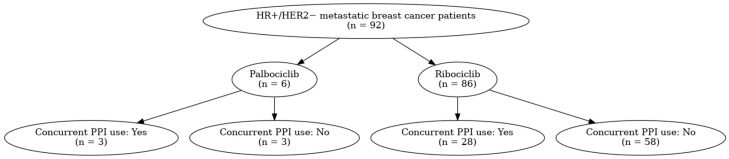
CDK4/6 inhibitor and PPI use in patients with HR+/HER2− metastatic breast cancer. HER2: human epidermal growth factor receptor 2, HR: hormone receptor, PPI: proton pump inhibitor, CDK4/6 inhibitor: cyclin-dependent kinase 4/6 inhibitor.

The study involved the enrollment of 92 patients with HR+/HER2− mBC. The median age at diagnosis was 59 years (minimum–maximum: 34–86). Visceral metastases were present in 56 patients (60.9%), and the majority—58 patients (63%)—were postmenopausal at the time of treatment initiation. Among them, 6 patients (6.5%) received palbociclib, while 86 patients (93.5%) were treated with ribociclib. A total of 31 patients (33.7%) received concomitant PPI during CDK4/6 inhibitor and endocrine therapy (Figure 1). Among the patients receiving palbociclib, 50.0% (*n* = 3) were concurrent PPI users, whereas among those receiving ribociclib, 32.6% (*n* = 28) were concurrent PPI users.

Of the included patients, 68 (73.9%) were classified as endocrine-sensitive, and 45 (48.9%) received CDK4/6 inhibitors as first-line therapy in the metastatic setting. As part of the endocrine therapy combined with CDK4/6 inhibitors, 36 patients (39.1%) received fulvestrant, while 55 patients (59.8%) were treated with aromatase inhibitors. A total of 27 patients (29.3%) receiving CDK4/6 inhibitors required dose reductions. Among those treated with ribociclib, 20 patients (21.7%) were reduced to a 400 mg dose and 4 patients to a 200 mg dose. Of the six patients receiving palbociclib, three (50%) required dose reductions, with two patients reduced to 100 mg and one patient to 75 mg. Table 1 demonstrates that the baseline clinical and therapeutic characteristics were well-balanced between patients who received PPIs and those who did not.

**Figure 2:**
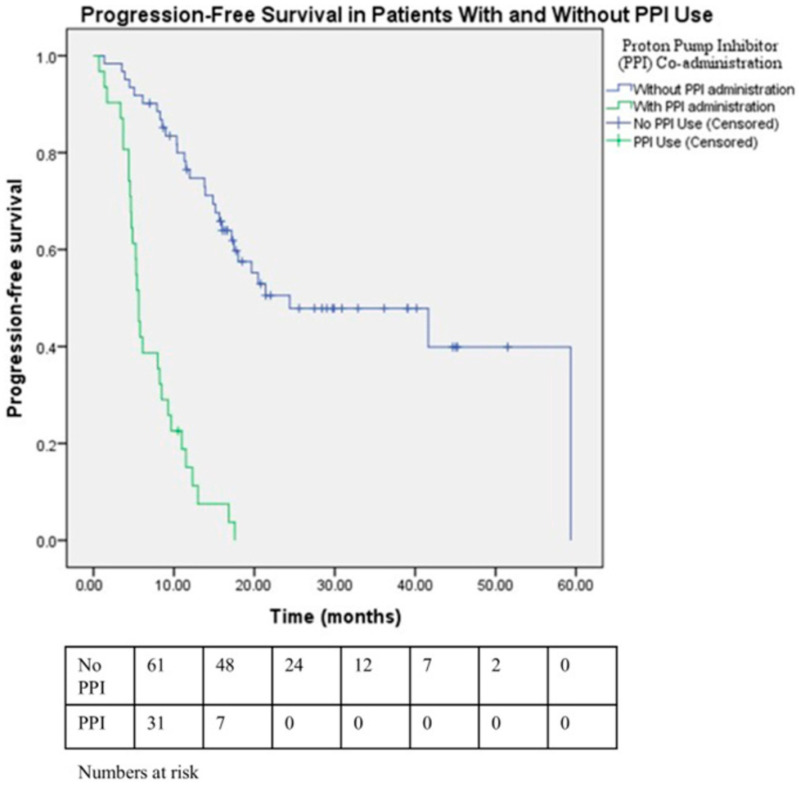
Kaplan–Meier curves illustrating PFS in patients treated with CDK4/6 inhibitors plus endocrine therapy, stratified according to concomitant PPI use. Patients not receiving PPIs are shown by the blue line, whereas the green line denotes those receiving PPIs. Tick marks (+) on the curves denote censored observations (patients without documented progression at their last follow-up or at the study cutoff). Downward steps indicate progression events. Numbers at risk are displayed below the x-axis.

The cohort had a median follow-up duration of 31.6 months. Several clinical parameters were examined in the univariate analysis for PFS, including age, ECOG status, menopausal status, visceral disease involvement, the total number of metastatic lesions, evidence of resistance to endocrine therapy, adjustments in dosing regimens, and concurrent PPI use. In patients receiving a combination of CDK4/6 inhibitors and ET, a significantly shorter PFS was observed in those who used concomitant PPI compared with non-users (median PFS: 5.6 months [95% CI: 5.05–6.19] vs. 24.4 months [95% CI: 4.44–44.38, *p* < 0.001]; Figure 2). A substantial difference in ORR was observed between PPI users and non-users, with rates of 25.8% and 57.4%, respectively (*p* = 0.004). Similarly, the DCR was substantially higher in patients without concomitant PPI use than in those with PPI use (85.2% vs. 38.7%, *p* < 0.001). The analysis revealed a significant association between poor performance status (ECOG ≥ 2) and shorter progression-free survival, with an estimated hazard ratio (HR) of 2.10 (95% CI: 1.03–4.31; *p* = 0.042). Patients with endocrine-resistant disease exhibited an approximately threefold increased risk of progression compared with those with endocrine-sensitive disease (HR: 2.92; 95% CI: 1.71–4.98; *p* < 0.001). Patients presenting with over three metastatic sites exhibited a markedly elevated risk of disease progression relative to those with fewer sites (HR = 2.65, 95% CI: 1.53–4.60, *p* < 0.001). Among patients with visceral metastases, the risk of disease progression was approximately 60% higher compared with those without visceral involvement (HR: 1.60; 95% CI: 0.93–2.77; *p* = 0.091); however, this association did not reach statistical significance. In the multivariable model incorporating key clinical factors, only three variables retained independent prognostic value for shorter progression-free survival: the presence of ≥3 metastatic sites, endocrine resistance, and concurrent use of proton pump inhibitors (Table 2).

When evaluated regarding treatment-related toxicity, dose reductions were required in nearly one-third of patients, with similar rates between groups (29.5% in the non-concomitant PPI group vs. 29.0% in the PPI group; *p* = 0.96). Likewise, dose interruptions occurred in 44.3% of patients without concomitant PPI use and in 45.2% of those with PPI use (*p* = 0.93). When comparing any cause grade ≥ 3 adverse events between groups, the statistical analysis revealed comparable results between patients with and those without concomitant PPI use (45.5% vs. 56.5%, respectively; *p* = 0.39). A similar incidence of grade ≥ 3 neutropenia was reported in patients treated with PPIs (47.6%) and those who did not receive PPIs (58.1%), with no statistical significance (*p* = 0.427).

## 4. Discussion

CDK4/6 inhibitors have become a cornerstone of treatment in HR+/HER2−mBC, particularly when used in combination with ET [22,24,25]. Their potential benefit is also being explored in early-stage disease, where they have demonstrated improvements in invasive disease-free survival (iDFS) [26,27]. Since these agents undergo hepatic metabolism predominantly via the CYP3A4 enzyme, they are vulnerable to drug–drug interactions, particularly in patients with polypharmacy due to comorbid conditions [28]. Notably, co-treatment with PPIs was found to be a significant and independent driver of progression, alongside resistance to endocrine therapy and a higher number of metastatic lesions in our study. The wide 95% CI around the non-PPI median PFS reflects heavy right-censoring with few progression events and not instability of the Kaplan–Meier curve. Because censoring limits the precision of median estimates, the non-PPI median should be interpreted with caution. To improve transparency, we report the numbers at risk and censoring patterns and provide fixed-time PFS estimates in the Supplementary Material.

Although there are several observational studies and meta-analyses in the literature, their results have yielded conflicting signals [14,29,30]. The potential impact of concomitant PPI use on the efficacy of CDK4/6 inhibitors remains a central and timely clinical question [31]. In a study conducted by Caglayan and colleagues, patients using PPIs during CDK4/6 inhibitor treatment had a median PFS of 10.9 months. Conversely, the median PFS was not reached in the group that did not use PPIs, indicating a significantly longer duration [29]. Supporting this, a meta-analysis by Moraes et al. demonstrated that concomitant use of PPIs with CDK4/6 inhibitors was associated with a twofold increase in the risk of disease progression (HR: 2.09; 95% CI: 1.41–2.95; *p* < 0.001) [14]. In a meta-analysis by Chang et al., concomitant use of PPIs with palbociclib was linked to a nearly twofold increase in the risk of mortality (HR = 2.03; 95% CI, 1.49–2.77; I^2^ = 0%), indicating a potentially detrimental effect on overall survival [32]. In contrast, no statistically significant association was observed between PPI coadministration and disease progression in patients treated with ribociclib. One suggested explanation for this differential impact involves the pharmacokinetic profile of palbociclib, particularly its minimum effective plasma concentration—commonly described by the ratio of the steady-state concentration to the in vitro IC_50_ (CSS/IC_50_)—which remains close to a critical threshold (approximately 0.94). This narrow margin implies that even modest reductions in drug absorption may impair its clinical efficacy. Ribociclib, on the other hand, appears to have a wider therapeutic window regarding the CSS/IC_50_ ratio, potentially reducing the clinical relevance of altered absorption due to concomitant PPI use [32]. However, the analysis had several limitations, including a small number of studies evaluating ribociclib—only four in total—and substantial heterogeneity between them.

A retrospective study by Odabas et al. reported no adverse impact of concurrent PPI use on PFS among patients treated with palbociclib (14.4 vs. 15.8 months, *p* = 0.82). They also reported no statistically significant impact of PPI use on PFS in ribociclib-treated patients (22.4 vs. 20.2 months, *p* = 0.40) [15]. Similarly, Takahashi et al. conducted a multicenter retrospective analysis and found no significant effect of PPIs on the efficacy of palbociclib or abemaciclib (HR for PFS = 1.19; 95% CI, 0.70–2.02) [33]. A more recent meta-analysis by Guo et al. concluded that PPI use did not significantly alter the risk of disease progression in patients receiving palbociclib (HR = 1.40; 95% CI, 1.07–1.84) [30]. In line with these findings, Del Re et al. observed no significant difference in PFS with ribociclib when used alongside PPIs (35.3 vs. 49.2 months, *p* = 0.594), attributing this to ribociclib’s higher pH tolerance and broader therapeutic index [16].

Differences in agent mix (palbociclib vs. ribociclib), exposure ascertainment (including unrecorded OTC PPI use and dosing timing), and confounding control likely explain much of the inconsistency across studies. Our ribociclib-dominant real-world results align with reports suggesting harm; however, given the risk of residual confounding—including protopathic and channeling bias—they should be interpreted as associational and require prospective agent-specific confirmation. Notably, the magnitude of the association between PPI co-administration and shorter PFS exceeds what might be expected from an absorption-only mechanism. Although sensitivity analyses (including time-dependent models, restrictions such as the ribociclib-only subset, and a negative-control analysis) consistently supported the direction of effect, unmeasured factors may still contribute to the observed size of the association. Protopathic bias may occur if PPIs are started for symptoms (e.g., dyspepsia, nausea) or for gastroprotection with steroids/NSAIDs that herald evolving disease, making PPI exposure a marker of worsening status. Channeling bias is also plausible if patients with greater comorbidity or clinical acuity are preferentially prescribed PPIs. We attempted to mitigate these biases through multivariable adjustment (ECOG, Charlson Comorbidity Index, polypharmacy, treatment line, metastatic burden, endocrine sensitivity) and a ribociclib-only restriction, but residual confounding (e.g., frailty, OTC PPIs, lifestyle) cannot be excluded. Taken together, these considerations support an associational interpretation and motivate prospective agent-specific pharmacokinetic studies that record exact dosing times and systematically capture confounders to gauge how much of the signal is causal. In practice, our findings argue for prudence rather than prohibition: minimize non-essential PPI use during CDK4/6 inhibitor therapy when reasonable alternatives exist (e.g., H_2_-receptor antagonists or short-course antacids), while individualizing decisions to the patient context. If a PPI is clinically unavoidable, one should use the lowest effective dose for the shortest duration, document the indication, provide counseling on separating administration in practice (staggered dosing where feasible), and monitor closely.

Our study has several limitations, including a relatively small sample size that precludes robust subgroup analyses for patients receiving palbociclib and the absence of patients treated with abemaciclib due to reimbursement restrictions in our country. Most patients were treated with ribociclib, and the retrospective design introduces potential sources of bias. In patients initiating PPIs after CDK4/6i start, the period before the first PPI day is intrinsically event-free among those ultimately classified as exposed. If counted within the exposed group, this “immortal” interval can attenuate a harmful association (bias toward the null or apparent benefit). Given that we still observed shorter PFS with PPIs, the effect size is unlikely to be inflated by “immortal” time and may be conservative. A key limitation is the absence of hour-level dosing data for PPIs and CDK4/6 inhibitors. Without documented administration times, we cannot determine whether patients took the agents concurrently or staggered them (e.g., separated by several hours). This uncertainty could plausibly attenuate a true interaction (if some patients routinely separated doses) or inflate it (if most took the drugs together) and, therefore, may influence the magnitude of the association we observed. Prospective agent-specific PK/PD studies that capture exact dosing times and test standardized staggering strategies are needed to quantify the extent to which timing modifies any pharmacokinetic interaction. Nevertheless, this work provides meaningful insights into a controversial area within the current literature by offering a comprehensive analysis from a tertiary referral center, supported by an extended follow-up period.

Unnecessary PPI prescriptions should be avoided, and alternative agents (like H_2_ receptor antagonists) may be preferred in patients who require gastroprotective therapy. Furthermore, it should be noted that the conflicting results in the literature may be due to heterogeneity in patient populations, treatment regimens, and the duration of PPI use. Therefore, prospective, multicenter, and drug-specific studies are needed to clearly define the interaction between PPIs and CDK4/6 inhibitors.

## 5. Conclusions

CDK4/6 inhibitors have transformed the care of HR+/HER2− metastatic breast cancer, producing meaningful survival gains. Whether PPIs diminish their effectiveness via pH-mediated pharmacokinetic effects remains uncertain: some meta-analyses and cohorts suggest reduced efficacy (notably for palbociclib and, in some settings, ribociclib), whereas others find no material association. In our cohort, concomitant PPI use was independently associated with a higher risk of progression. Because the population was ribociclib-dominant, these findings primarily apply to ribociclib and should not be generalized to other CDK4/6 inhibitors without confirmatory data. Importantly, the magnitude of the association must be interpreted in light of potential protopathic bias and channeling bias. Despite multivariable adjustment, such residual confounding may persist and limits causal inference. Until prospective agent-specific data are available, we recommend cautious use—minimizing non-essential PPI therapy when feasible—and further study to clarify causality and dosing strategies.

## Figures and Tables

**Table 1:** Baseline clinicopathological and treatment characteristics of the patients.

	Total (*n* = 92)	Concomitant Use of PPI		*p*-Value
		Yes (*n* = 31)	No (*n* = 61)	
Age (years), median (range)	59 (34–86)	60 (34–84)	58 (36–86)	0.77
ECOG PS, *n* (%)				
0	33 (35.9)	10 (32.3)	23 (37.7)	0.34
1	50 (58.7)	16 (51.6)	34 (55.7)	
2–3	9 (7.6)	5 (16.1)	4 (6.6)	
CDK4/6 inhibitor type, *n* (%)				
Ribociclib	86 (93.5)	28 (90.3)	58 (95.1)	0.36
Palbociclib	6 (6.5)	3 (9.7)	3 (4.9)	
Charlson Comorbidity Index (CCI)				
CCI ≤ 7	48 (52.2)	16 (51.6)	32 (52.5)	1.00
CCI > 7	44 (47.8)	15 (48.4)	29 (47.5)	
Polypharmacy				
<5 agents	75 (81.5)	24 (77.4)	51 (83.6)	0.661
≥5 agents	17 (18.5)	7 (22.6)	10 (16.4)	
Menopausal status, *n* (%) *				
Premenopausal	34 (37.0)	10 (32.3)	24 (39.3)	0.48
Postmenopausal	58 (63.0)	21 (67.7)	37 (60.7)	0.48
Concomitant LH–RH agonist, *n* (%)				
None	69 (75)	22 (71)	47 (77)	0.09
Goserelin acetate	23 (25)	9 (29)	14 (23)	
Concomitant endocrine therapy, *n* (%)				
Anastrozole	4 (4.3)	4 (12.9)	0 (0.0)	0.43
Exemestane	3 (3.3)	2 (6.5)	1 (1.6)	
Letrozole	49 (53.2)	13 (41.9)	36 (59.0)	
Fulvestrant	36 (39.1)	12 (38.7)	24 (39.3)	
Endocrine sensitivity status of the disease, *n* (%)				
Sensitive	68 (73.9)	21 (67.7)	47 (77)	0.33
Resistant	24 (26.1)	10 (32.3)	14 (23)	
Type of disease, *n* (%)				
De novo	44 (47.8)	12 (38.7)	32 (52.5)	0.21
Recurrent	48 (52.2)	19 (61.3)	29 (47.5)	
Treatment line, *n* (%)				
1st	45 (48.9)	10 (32.3)	35 (57.4)	0.057
2nd	25 (27.2)	10 (32.3)	15 (24.6)	
≥3rd	22 (23.9)	11 (35.5)	11 (18)	
Metastatic pattern, *n* (%)				
Visceral involvement †	56 (60.9)	20 (64.5)	36 (59.0)	0.60
Non-visceral	36 (39.1)	11 (35.5)	25 (41)	
Lung	39 (42.4)	13 (41.9)	26 (42.6)	0.95
Liver	23 (25)	9 (29)	14 (23)	0.52
Lung+ liver	10 (10.9)	3 (9.7)	7 (11.5)	0.79
Bone lesion only	31 (33.7)	10 (32.3)	21 (34.4)	0.83
Number of metastatic sites, *n* (%)				
<3	63 (68.5)	18 (58.1)	45 (73.8)	0.12
≥3	29 (31.5)	13 (41.9)	16 (26.2)	
Dose reduction, *n* (%)	27 (29.3)	9 (29)	18 (29.5)	0.96
Ribociclib 400 mg	20 (21.7)	6 (19.4)	14 (23)	
Ribociclib 200 mg	4 (4.3)	1 (3.2)	3 (4.9)	
Palbociclib 100 mg	2 (2.2)	1 (3.2)	1 (1.6)	
Palbociclib 75 mg	1 (1.1)	1 (3.2)	0 (0.0)	
PPI used, *n* (%)				
Esomeprazole		6 (19.4)		
Lansoprazole		12 (38.7)		
Pantoprazole		13 (41.9)		

**Abbreviations:** ECOG PS, Eastern Cooperative Oncology Group (ECOG) performance status; PPI, proton pump inhibitor. * In the ribociclib group, three patients were male. † Cases with metastatic spread to the lungs, liver, brain, pleural surfaces, or the peritoneal cavity were categorized as having visceral metastases.

**Table 2:** Univariate and multivariate Cox regression analysis for progression-free survival.

	Univariate		Multivariate	
	HR (95%CI)	*p*-Value	HR (95%CI)	*p*-Value
Age (years)				
≤59 (ref)	0.76 (0.45–1.27)	0.29	1.49 (0.63–3.49)	0.364
>59				
ECOG PS				
ECOG 0–1 (ref)	2.1 (1.03–4.31)	**0.042**	1.25 (0.48–3.22)	0.651
ECOG ≥ 2				
Charlson Comorbidity Index (CCI)				
CCI > 7 (ref)	1.21 (0.72–2.02)	0.47	0.74 (0.33–1.66)	0.463
CCI ≤ 7				
Polypharmacy				
≥5 chronic agents	1.07 (0.57–2.01)	0.84	1.66 (0.73–3.78)	0.230
<5 agents				
Menopausal status				
Premenopausal (ref)	1.36 (0.71–2.63)	0.35	1.02 (0.39–2.60)	0.968
Postmenopausal				
Endocrine sensitivity status of the disease				
Sensitive (ref)	2.92 (1.71–4.98)	**<0.001**	2.50 (1.34–4.65)	**0.004**
Resistant				
Metastatic pattern				
Non-visceral (ref)	1.60 (0.93–2.77)	0.091	0.59 (0.26–1.31)	0.198
Visceral involvement				
Number of metastatic sites				
<3 (ref)	2.65 (1.53–4.60)	**<0.001**	3.06 (1.39–6.76)	**0.005**
≥3				
Treatment line				
1 L (ref)				
2 L	2.11 (1.15–3.86)	**0.015**	2.04 (0.95–4.39)	0.069
≥3 L	2.42 (1.27–4.63)	**0.007**	2.51 (1.12–5.63)	**0.026**
Dose reduction				
No (ref)	0.78 (0.44–1.39)	0.40	0.62 (0.33–1.18)	0.147
Yes				
Concomitant use of PPI				
No (ref)	7.26 (4.04–13.06)	**<0.001**	6.82 (3.33–13.96)	**<0.001**
Yes				

**Abbreviations:** ECOG PS, Eastern Cooperative Oncology Group (ECOG) performance status; PPI, proton pump inhibitor. Variables with statistical significance are indicated in bold. (ref) indicates reference category.

## Data Availability

The data presented in this study are available on reasonable request from the corresponding author. The data are not publicly available due to privacy and ethical restrictions.

## Supplementary Materials

The following supporting information can be downloaded at: https://www.mdpi.com/article/10.3390/medicina61111960/s1.

## Abbreviations

The following abbreviations are used in this manuscript:

AI	Aromatase inhibitor
CDK4/6	Cyclin-dependent kinase 4/6
CDK4/6i	Cyclin-dependent kinase 4/6 inhibitor(s)
CI	Confidence interval
CTCAE	Common Terminology Criteria for Adverse Events
CYP3A4	Cytochrome P450 3A4
DCR	Disease control rate
ECOG PS	Eastern Cooperative Oncology Group Performance Status
ER	Estrogen receptor
ET	Endocrine therapy
GERD	Gastroesophageal reflux disease
HER2	Human epidermal growth factor receptor 2
HR	Hazard ratio
HR+/HER2	Hormone receptor–positive/HER2-negative
IHC	Immunohistochemistry
ISH	In situ hybridization
iDFS	Invasive disease-free survival
LHRH	Luteinizing hormone–releasing hormone
mBC	Metastatic breast cancer
ORR	Overall response rate
OS	Overall survival
OTC	Over-the-counter
PFS	Progression-free survival
PPI	Proton pump inhibitor
PK/PD	Pharmacokinetics/pharmacodynamics
SPSS	Statistical Package for the Social Sciences
C_ss/IC50	Steady-state drug concentration to half-maximal inhibitory concentration ratio
