# Supplementary material for: Assessing the Influence of Proton Pump Inhibitors on Clinical Outcomes in Hormone Receptor-Positive Metastatic Breast Cancer Patients Receiving CDK4/6 Inhibitors: Evidence from a Ribociclib-Dominant Cohort
Source: Medicina (Kaunas). 2025 Oct 31;61(11):1960. doi: 10.3390/medicina61111960 (PMC12654086; doi:10.3390/medicina61111960)

Table S1. Baseline Clinicopathological and Treatment Characteristics of Ribociclib-treated Patients (n=86) by Concomitant PPI Use

Characteristic	Level	Total (n=86)	Yes (n=28)	No (n=58)	p-value
Age (years), median (range)		60 (35-86)	59 (37-76)	60 (35-86)	0.84
ECOG PS, n (%)	0	28 (32.6%)	7 (25%)	21 (36.2%)	0.402
	1	49 (57%)	16 (57.1%)	33 (56.9%)	
	2	7 (8.1%)	4 (14.3%)	3 (5.2%)	
	3	2 (1.7%)	1 (3.6%)	1 (1.7%)	
Charlson Comorbidity Index (CCI)	CCI ≤7	44 (51.2%)	13 (46.4%)	31 (53.4%)	0.704
	CCI >7	42 (48.8%)	15 (53.6%)	27 (46.6%)	
Polypharmacy	<5 agents	70 (81.4%)	22 (78.6%)	48 (82.8%)	0.864
	≥5 agents	16 (18.6%)	6 (21.4%)	10 (17.2%)	
Menopausal status, n (%)	Premenopausal	18 (21.2%)	5 (18.5%)	13 (22.4%)	0.682
	Postmenopausal	67 (78.8%)	22 (81.5%)	45 (77.6%)	
Endocrine Sensitivity Status of the Disease, n (%)	Sensitive	64 (74.4%)	19 (67.9%)	45 (77.6%)	0.333
	Resistant	22 (25.6%)	9 (32.1%)	13 (22.4%)	
Treatment line, n (%)	1st	42 (48.8%)	8 (28.6%)	34 (58.6%)	0.021
	2nd	23 (26.7%)	9 (32.1%)	14 (24.1%)	
	≥ 3rd	21 (24.4%)	11 (39.3%)	10 (17.2%)	
Metastatic Pattern, n (%)	Visceral Involvement	51 (59.3%)	18 (64.3%)	33 (56.9%)	0.675
	Non-Visceral	35 (40.7%)	10 (35.7%)	25 (43.1%)	
Number of metastatic sites, n (%)	<3	60 (69.8%)	16 (57.1%)	44 (75.9%)	0.077

	≥3	26 (30.2%)	12 (42.9%)	14 (24.1%)	
Dose reduction, n (%)	No	63 (73.3%)	21 (75.0%)	42 (72.4%)	1.000
	Yes	23 (26.7%)	7 (25.0%)	16 (27.6%)	

Table S2. Multivariable Cox Regression for Progression-Free Survival — Ribociclib subgroup

Covariate	Adjusted HR (95% CI)	p-value
**Age >59 vs ≤59**	1.56 (0.65–3.74)	0.320
**CCI ≤7 vs >7**	0.73 (0.30–1.75)	0.478
**Polypharmacy ≥5 vs <5**	1.95 (0.81–4.67)	0.135
**Dose reduction (Yes vs No)**	0.63 (0.32–1.26)	0.194
**Concomitant PPI (Yes vs No)**	**6.36 (3.02–13.37)**	**<0.001**
**Treatment line**		**0.019** (overall)
└ 2L vs 1L	**2.41 (1.04–5.56)**	**0.039**
└ ≥3L vs 1L	**3.19 (1.37–7.43)**	**0.007**
**Endocrine resistance (Yes vs Sensitive)**	**2.21 (1.15–4.24)**	**0.006**
**Postmenopausal vs Premenopausal**	1.03 (0.37–2.84)	0.958
**Number of metastatic sites ≥3 vs <3**	**2.96 (1.28–6.86)**	**0.011**
**Visceral involvement (Yes vs No)**	0.56 (0.27–1.14)	0.112
**ECOG ≥2 vs 0–1**	1.48 (0.73–3.02)	0.287

Figure S1. Kaplan–Meier curves for progression-free survival (PFS) by concomitant PPI use in the ribociclib subgroup (n=86).

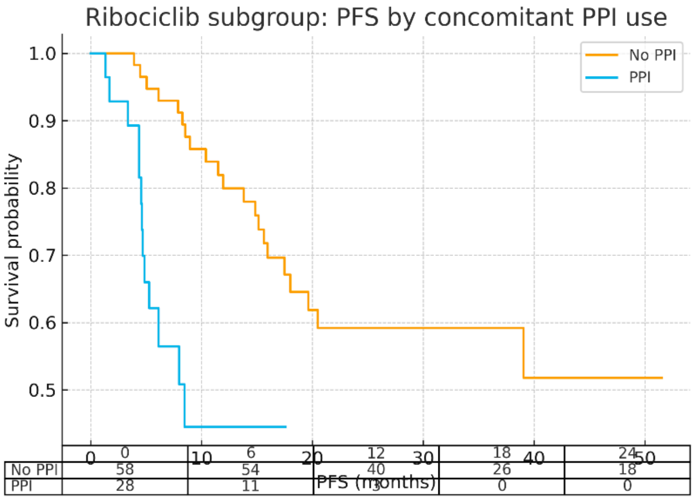

Kaplan–Meier estimates of progression-free survival (PFS) among patients treated with ribociclib, stratified by concomitant proton-pump inhibitor (PPI) use. The table below the plot shows numbers at risk at 0, 6, 12, 18, and 24 months. Median PFS (months) with 95% confidence intervals (CIs) were 21.39 (4.33–38.44) for the No PPI group and 5.39 (4.92–5.86) for the PPI group. The overall difference in survival distributions was significant by log-rank (Mantel–Cox) test: χ^2=54.87, df=1, p<0.001.

Figure notes: Lines are stepwise KM estimates; censored observations are marked with ticks. The No PPI group shows limited events beyond the median, yielding a wide CI.

In the ribociclib subgroup (n=86), median PFS was 21.4 months without concomitant PPIs versus 5.4 months with PPIs (log-rank χ^2=54.9, p<0.001). Findings were concordant with multivariable Cox modeling in this subgroup, which retained a strong adverse association for PPI co-administration after adjustment for clinical covariates (see Table S2).

Figure S2. Cumulative number of progression events over time by group. Lines depict stepwise accumulation of events (months since CDK4/6 inhibitor start).

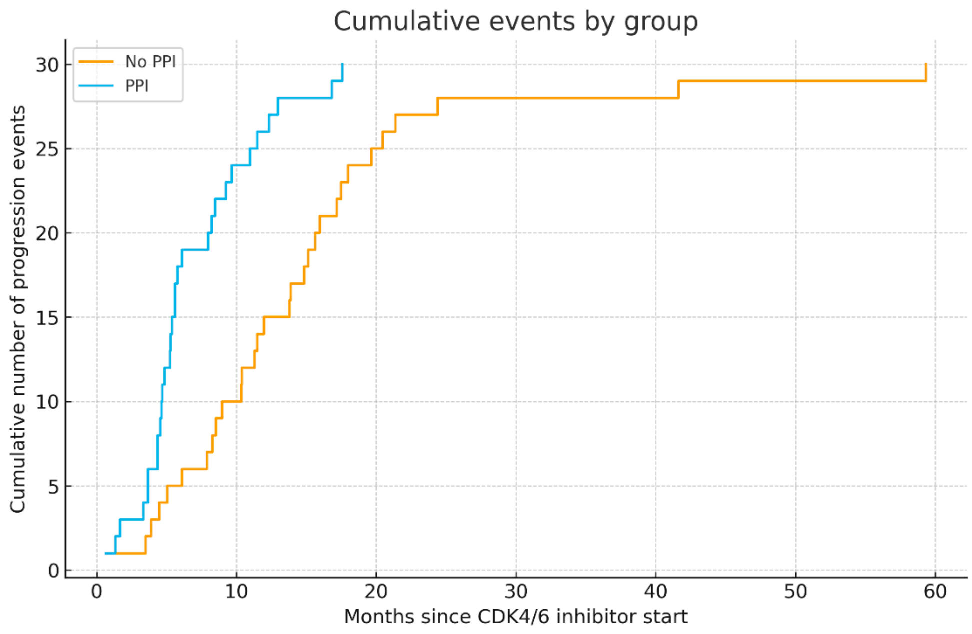

Figure S3. Fixed-time PFS probabilities at 6, 12, 18, and 24 months. Bars show KM estimates with Greenwood 95% CIs

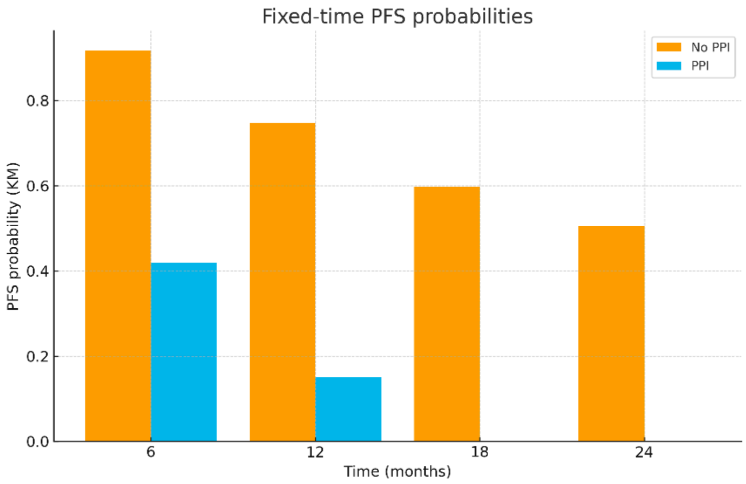

Supplement: Supplementary file 1 [file medicina-61-01960-s001.zip › medicina-3898432-supplementary.pdf]
